# Histopathological and Clinical Features of HER2-Positive Breast Cancers across Hormone Receptor Subgroups: A Cross-Sectional Analysis

**DOI:** 10.12688/f1000research.171847.1

**Published:** 2025-11-13

**Authors:** Anuradha Calicut Kini Rao, Kanthilatha Pai, Swathi Prabhu, Naurin Kasem, Karthik S Udupa

**Affiliations:** 1 Department of Pathology, Kasturba Medical College Manipal, Manipal Academy of Higher Education, Manipal, Karnataka, 576104, India; 2 Department of Medical Oncology, Kasturba Medical College Manipal, Manipal Academy of Higher Education, Manipal, Karnataka, 576104, India

**Keywords:** breast carcinoma, HER2-positive, HER2-enriched, Luminal B

## Abstract

**Background:**

HER2-positive breast carcinomas are a heterogeneous group with various clinicopathological characteristics depending on hormone receptor (HR) status. This study aimed to analyze and compare the clinical and histopathological features of HER2-positive breast carcinomas across three subgroups: Group 1: Triple-positive (ER+/PR+/HER2+), Group 2:ER-positive only (ER+/PR-/HER2+), and Group 3: HER2-enriched (ER-/PR-/HER2+).

**Methods:**

This was a single-institution, retrospective, cross-sectional study conducted over 42 months (January 2021–June 2024) at a tertiary care center comprising of 117 HER2-positive breast carcinoma cases. The demographic, clinical, and histopathological parameters were studied retrospectively. Statistical analysis was performed via SPSS Version 29.0, employing ANOVA and chi-square tests, with p < 0.05 considered significant.

**Results:**

Among 117 HER2-positive breast cancer patients, left and right breast involvement was nearly equal. Most were diagnosed via core biopsy (83.8%), with the upper outer quadrant most commonly affected (42.9%). Invasive breast carcinoma of no special type was the predominant subtype. The HER2-enriched group (Group 3) had the highest mean age (57.28 years; *p* = 0.001) and the most Grade 3 tumors (36.1%; *p* = 0.005). Although Group 2 (ER-positive) had the largest mean tumor size (4.31 cm), the difference was not statistically significant ( *p* = 0.143). Lymphovascular invasion, necrosis, Ductal carcinoma in situ (DCIS), and skin involvement showed no significant differences across groups. ANOVA showed significant variation in age and grade, and post-hoc analysis confirmed that Group 1 vs Group 3 differed significantly in both age ( *p* = 0.001) and grade ( *p* = 0.004).

**Conclusion:**

HER2-positive breast carcinomas show variability by HR status. The HER2-enriched subtype is associated with older age, higher histological grade, and advanced nodal stage, suggesting a more aggressive phenotype. Recognizing these patterns aids in accurate prognostication and guiding individualized therapy.

## Introduction

Breast cancer ranks as the most prevalent cancer among women globally, accounting for approximately 25% of all female cancer cases. In India, it holds the top position among cancers affecting women, constituting approximately 28.2% of all female cancers. By 2022, approximately 216,000 new cases are projected to be diagnosed across the country. ^1, 2^ Luminal A breast cancers are traditionally believed to have a favorable prognosis, whereas triple-negative breast cancers (TNBCs) are associated with poor outcomes. However, clinical observations revealed that some Luminal A tumors exhibited aggressive behavior, whereas certain TNBC cases presented relatively better outcomes. These inconsistencies led to the exploration and development of more refined molecular subtyping of breast cancer. ^3^ Among all breast cancer subtypes, HER2-positive tumors have drawn particular attention because of the availability of targeted therapy with trastuzumab. As a result, HER2-enriched cancers have been extensively studied in literature. In recent years, the classification has further evolved with the recognition of HER2-low and HER2-ultralow tumors, driven by the development of novel antibody–drug conjugates such as trastuzumab deruxtecan. ^4^ HER2 status in breast cancer is determined according to the ASCO/CAP guideline criteria via immunohistochemistry (IHC) and in situ hybridization (ISH). A positive HER2 IHC score is defined as 3+, which indicates complete, intense, circumferential membranous staining in more than 10% of the tumor cells. This staining should be easily visible at low magnification and should be observed in a homogenous and contiguous population of invasive tumor cells. A patient is considered HER2-positive if the IHC score is 3+ or if the IHC score is 2+ (equivocal) with a positive HER2 ISH result. Additionally, a tumor is classified as HER2 positive if HER2 ISH is positive, regardless of the IHC result. ^1^ Approximately 20–25% of breast cancers are HER2-positive, and among these, nearly half also express hormone receptor (HR). A growing body of research indicates that the clinical characteristics, biological behavior, treatment response, and prognosis of HER2-positive breast cancers are influenced by their HR status. ^5^ HR-positive HER2-positive breast tumors are associated with better survival outcomes than HR-negative HER2-positive tumors. Tumors that are positive for either the estrogen receptor (ER) or progesterone receptor (PR) alone tend to behave differently from those that are dual-positive (ER and PR) or dual-negative, indicating biological heterogeneity within HER2-positive subtypes. ^6– 8^ We conducted a retrospective cross-sectional study on HER2-positive breast carcinoma patients diagnosed over a period of 42 months at a tertiary care hospital. The patients were divided into three groups on the basis of HR status: group 1: triple-positive (ER+, PR+, HER2+), group 2: ER-positive only (ER+, PR−, HER2+), and group 3: HER2-enriched (ER−, PR−, HER2+). The objective of this study was to evaluate and compare the clinicopathological features across these molecular subtypes. Our findings provide insights into the biological behavior and morphological spectrum of HER2-positive breast cancers in relation to HR expression.

## Materials and methods

**Figure 1.**
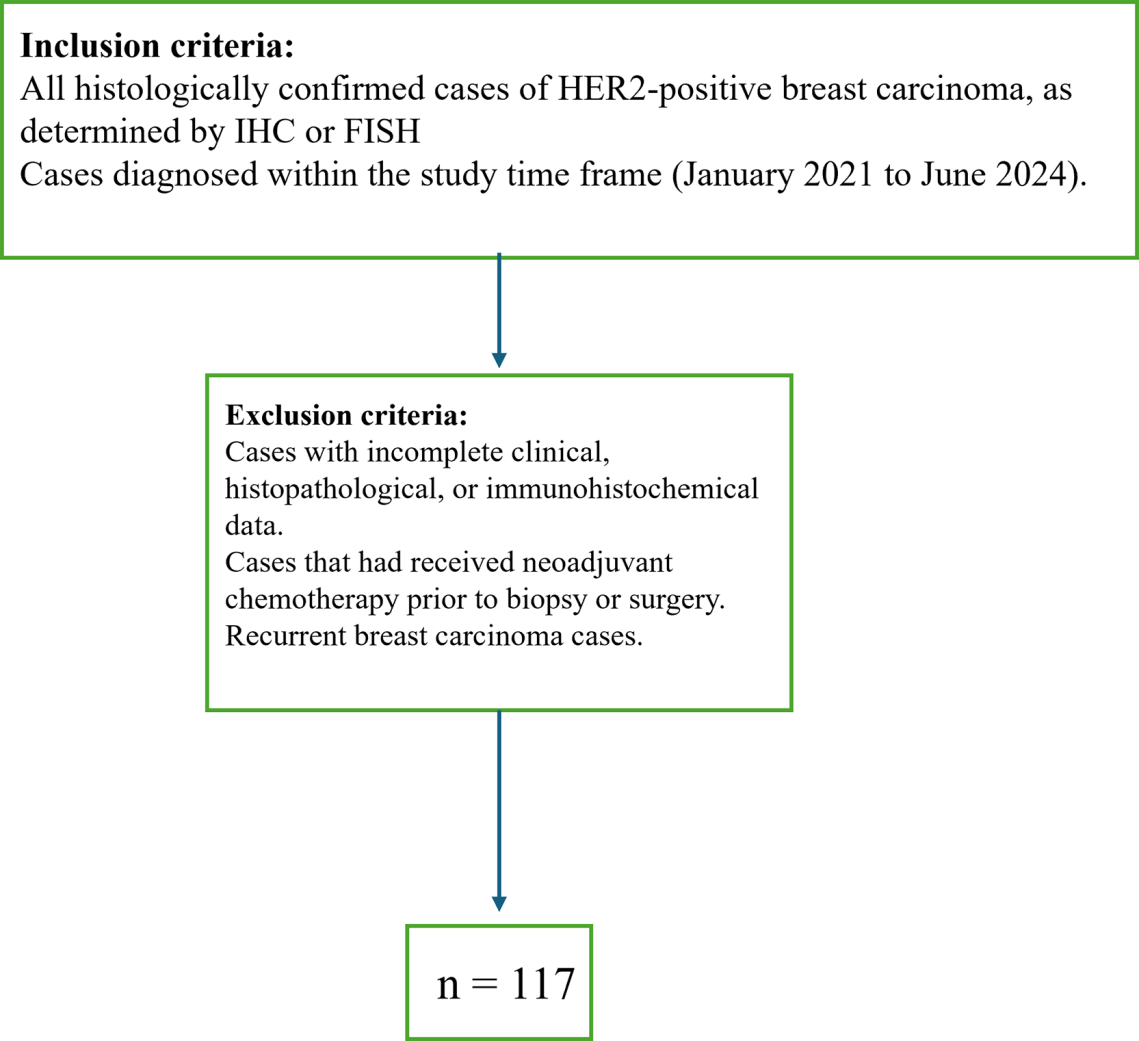
Methodology with inclusion and exclusion criteria. The total cases included in our study were 117.

This was a single institutional, cross-sectional, retrospective observational analysis involving cases received at the pathology laboratory of a tertiary care hospital for a duration of 42 months (January 2021-June 2024). After applying the relevant inclusion and exclusion criteria ( Figure 1), 117 patients were included in the study group. Patients whose HER2- status was negative/those whose clinicopathological data were insufficient, or whose IHC status was unknown were excluded. The demographic data included the age of the patient, the location within the breast, and the laterality; the morphological data included the size of the tumor, the histopathologic grade, lymphovascular invasion (LVI), perineural invasion (PNI), necrosis, ductal carcinoma in situ (DCIS), skin involvement, TNM stage and Ki67. Clinical TNM staging was used when only core biopsies were available, and pathological TNM staging was used for breast conserving surgery (BCS) and modified radical mastectomy (MRM) cases. Molecular subtyping was performed based on the IHC assessment of HR status and HER2 positivity, and the latter was tested via the ISH technique in a reference laboratory in cases in which HER2 was equivocal on IHC.

### Patients

A total of 117 newly diagnosed HER2-positive breast carcinoma cases diagnosed between January 2021 and June 2024, spanning a period of 42 months, were studied. HER2 positivity was defined based on the ASCO/CAP guidelines as either IHC 3+ or with ISH amplification.

The inclusion and exclusion criteria were employed for selection, and the cases were further divided into three study groups according to hormone receptor status ( Table 1).

**Table 1.** The table shows three study groups according to hormone receptor status.

Groups	Estrogen receptor (IHC)	Progesterone receptor (IHC)
Group 1 (TP)	Positive	Positive
Group 2 (ER-positive only)	Positive	Negative
Group 3 (HER2-enriched)	Negative	Negative

TP, Triple-positive; IHC, Immunohistochemistry.

### Statistics

SPSS Statistics for Windows, Version 29.0. (Armonk, NY: IBM Corp.). To describe the data, descriptive statistics, frequency analysis, and percentage analysis were used for categorical variables, and the mean and SDs were used for continuous variables. To identify significant differences in the multivariate analysis, one-way ANOVA was used. To determine the significance of the differences in qualitative categorical data the chi-square test was used. For both of the above statistical tools, a probability value of 0.05 is considered significant.

## Results

A total of 117 HER2-positive breast carcinomas were analyzed. Among the 117 patients, 59 (50.4%) involved the left breast, and 58 (49.6%) involved the right breast, showing an almost equal distribution between the two sides. The tumors were located in the upper outer quadrant in 50 patients, accounting for 42.9% of the total. The upper inner quadrant was involved in 23 patients (19.6%), whereas the lower outer quadrant was affected in 19 patients (16.2%). The lower inner quadrant was involved in 11 patients (9.4%), and the central quadrant was involved in 8 patients (6.8%). Tumors involving all quadrants were observed in 6 patients, accounting for 5.1% of the total. The majority of patients (83.8%) were diagnosed with core biopsy. BCS accounted for 8.6% of the samples, whereas MRM accounted for 7.6% of the samples.

The study cohort of HER2-positive breast carcinoma was divided into three groups on the basis of HR status. Group 1 (Triple-positive) included 53 patients who were positive for both ER and PR. Group 2 (ER-positive only) comprised 28 patients who were positive for ER, negative for PR. Group 3 (HER2-enriched) consisted of 36 patients who were negative for both ER and PR. When the subgroup analysis was performed across the three groups, the mean age was significantly greater in the HER2-enriched group (57.28 years) than in the other groups (p = 0.001). The largest tumor size was detected in the ER-positive only group (4.31 cm), although this difference was marginally significant statistically (p = 0.143). The T2 stage was the most common stage across all groups, with a greater proportion in the HER2-enriched group (58.3%) (p = 0.061). Nodal involvement (N1) was more common in the HER2-enriched group (50%), but the difference was not significant (p = 0.266) ( Table 2).

**Table 2.** Clinical characteristics of HER2-enriched patients across various groups (n = 117).

Clinical parameters	Luminal B		HER2-enriched
	Group 1, n = 53 (Triple-positive)	Group 2, n = 28 (ER-positive only)	Group 3, n = 36 (HER2-enriched)
Age, y * (p = 0.001)	48.98 (9.20)	52.61 (10.51)	57.28 (10.86)
Laterality (p = 0.707)			
Right, n (%)	27 (50.9)	12 (42.9)	19 (52.8)
Left, n (%)	26 (49.1)	16 (57.1)	17 (47.2)
Tumor size, cm (p = 0.143)	3.34 (2.38)	4.31 (2.51)	3.42 (1.50)
Tumor stage (p = 0.061)			
T1, n (%)	13(24.5)	1 (3.6)	4 (11.1)
T2, n (%)	18 (34)	11 (39.3)	21 (58.3)
T3, n (%)	13 (24.5)	9 (32.1)	5 (13.9)
T4, n (%)	9 (17)	7 (25)	6 (16.7)
Nodal stage (p = 0.266)			
N0, n (%)	25 (47.2)	12 (42.9)	13 (36.1)
N1, n (%)	20 (37.7)	10 (35.7)	18 (50.0)
N2, n (%)	7 (13.2)	4 (14.3)	1 (2.8%)
N3, n (%)	1 (1.9%)	2 (7.1%)	4 (11.1)

* Values are expressed as the mean ± SD or median with range, unless otherwise specified. ER, Estrogen receptor.

When histopathological features were analyzed among the three groups, the predominant subtype across all groups was invasive breast carcinoma, not otherwise specified (IBC NOS), which was observed in 96.2% of the patients in group 1 (Triple-positive), 92.8% of those in group 2 (ER-positive only), and 88.9% of those in group 3 (HER2-enriched). Rare histological variants included micropapillary and mucinous features in group 1, medullary features in group 2, and apocrine/apocrine-like features in group 3 ( Table 3). The SBR grade distribution showed statistically significant difference (p = 0.005), with group 1 showing a predominance of grade 2 tumors (62.3%), group 2 having the highest proportion of grade 2 tumors (92.9%) and very few grade 3 tumors, and group 3 having the highest proportion of grade 3 tumors (36.1%). Other features, such as LVI, PNI, associated DICS, necrosis, and skin involvement, did not differ significantly between the groups.

**Table 3.** Histopathological features of patients across various groups (n = 117).

	Group 1(n = 53)	Group 2(n = 28)	Group 3(n = 36)	p-value
Histopathological subtypes				
Apocrine breast carcinoma, n (%)	0	0	1 (2.8)	
IBC with apocrine features, n (%)	0	0 (0)	3 (8.3)	
IBC with medullary features, n (%)	0	1 (3.6)	00 (0%)	
IBC with micropapillary features, n (%)	1 (1.9)	1 (3.6)		
IBC with mucinous differentiation, n (%)	1 (1.9)	0	0	
IBC NOS, n (%)	51 (96.2)	26 (92.8)	32 (88.9)	
SBR grade				0.005
Grade 1, n (%)	13 (24.5)	0 (0)	3 (8.3)	
Grade 2, n (%)	33 (62.3)	26 (92.9)	20 (55.6)	
Grade 3, n (%)	7 (13.2)	2 (7.1)	13 (36.1)	
LVI present, n (%)	9 (17)	6 (21.4)	6 (16.7)	0.859
PNI present, n (%)	3 (5.7)	0 (0)	4 (11.1)	0.176
DCIS, n (%)	14 (26.4)	6 (21.4)	10 (27.8)	0.834
Necrosis, n (%)	14 (26.4)	7 (25)	11 (30.6)	0.866
Skin Involvement, n (%)	10 (18.9)	7 (25)	6 (16.7)	0.694
Ki67 proliferation index, (mean) *	43.42 (23.5)	44.93 (22.94)	50.19 (23.80)	0.4

IBC NOS, Invasive breast carcinoma of no special type; LVI, lymphovascular invasion; PNI, perineural invasion; DCIS, ductal carcinoma in situ.

* Values are expressed as the mean ± SD or median with range, unless otherwise specified.

**Table 4.** Summary of ANOVA and post hoc Pairwise Comparisons for Clinicopathological Variables Among the three groups of HER2 -positive breast carcinomas.

Variable	ANOVA p value	Post hoc Group Comparison	Mean Difference (I–J)	p value	95% Confidence Interval
Age	0.001	group 1 vs group 2	-3.626	0.274	-9.202 to 1.950
		group 1 vs group 3	-8.2966	0.001	-13.452 to -3.142
		group 2 vs group 3	-4.6706	0.16	-10.685 to 1.344
SBR grade	0.004	group 1 vs group 2	-0.5256	0.081	-1.101 to 0.050
		group 1 vs group 3	-0.728	0.004	-1.260 to -0.196
		group 1 vs group 3	-0.2024	0.719	-0.823 to 0.418

When ANOVA was performed, statistically significant differences were observed in the mean age (p = 0.001) and SBR score (p = 0.004) among the three groups. However, there was no significant difference in tumor size (p = 0.143) or the Ki67 index (p = 0.400) between the groups. Post hoc analysis revealed a significant difference in age between group 1 and group 3 (p = 0.001), with group 3 being older. Similarly, SBR scores were significantly higher in group 3 than in group 1 (p = 0.004). No significant differences were observed between the other group pairs for either variable ( Table 4).

## Discussion

HER2-positive breast cancers are driven by amplification of the HER2 gene located on chromosome 17q. The HER2 protein is a receptor tyrosine kinase that activates signaling pathways such as the RAS and PI3K-AKT pathways, promoting tumor growth and survival. These tumors may or may not express hormone receptors. In addition to gene amplification, they often exhibit complex chromosomal alterations and a high number of genetic mutations. The differences in gene expression profiles among these tumors are largely determined by hormone receptor status and the expression of estrogen related genes. ^9^ HER2 status can be identified via IHC or ISH. Although HER2-positive cancers were once associated with poor prognosis, the development of targeted therapies has significantly improved outcomes, with more than half of patients now achieving remission. Nonetheless, resistance to these therapies can occur, and new treatment strategies are actively being explored. ^10, 11^ In the present study, IBC,NST was the most frequent histologic subtype across all groups, which is consistent with prior literature describing it as the predominant morphology in HER2-positive tumors. ^12^ A review of the available literature revealed increased HER2 expression in grade 2/grade 3 tumors, apocrine carcinomas (51%) and apocrine-like carcinomas (47%). ^13, 14^ Akashi et al. reported that non-Luminal HER2 (NLH- or HER2-enriched) patients frequently presented with comedonecrosis, whereas Luminal HER2 patients tended to have more central scarring. Moreover, NLH patients presented higher rates of tumor-infiltrating lymphocytes and demonstrated greater healing capabilities than Luminal B patients did. These histopathological distinctions and differences in immune responsiveness highlight the complexity and diversity of HER2-positive breast cancer subtypes. ^15^ Taucher et al. reported a significant correlation between HER2 overexpression and ER and PR receptor-negative status. Additionally, they reported greater HER2 overexpression in SBR grade 3 lesions and younger patients. Notably, in individuals with positive ER and PR status and with SBR grade 1/grade 2 disease, the likelihood of HER2-positivity was estimated to be approximately 6.1%. ^16^ A study by Omranipour reported that HER2-positivity was associated with larger tumor size and PR negativity, indicating more aggressive tumor characteristics. Similarly, in our study, HR-negative/HER2-positive tumors also presented greater proliferation (Ki67) and more high-grade histology than HR-positive/HER2-positive tumors did. Both studies highlight the link between hormone receptor negativity, particularly PR loss, and aggressive features in HER2-positive breast cancers. However, while Omranipour focused on predictors of HER2 positivity in a broader breast cancer population, our study specifically compared clinicopathological characteristics within HER2-positive patients on the basis of HR status. These findings consistently support the heterogeneity of HER2-positive breast cancers related to hormone receptor expression. ^17^ Cong et al. reported that the majority of breast cancers were invasive carcinomas of no special type, mostly grade 2, with a tumor size >2 cm and frequent lymph node involvement and that HER2 amplification or overexpression was associated with greater Ki67, larger tumor size, higher grade, stage, and the Nottingham Prognostic Index. In comparison, our study similarly revealed invasive carcinoma as the predominant histology, with HR-negative/HER2-positive tumors showing higher histological grade and Ki67 levels, reflecting more aggressive tumor biology. Both studies demonstrated a clear link between HER2 positivity and markers of tumor proliferation and aggressiveness. ^18^ Tamaki et al. reported that HER2-positive tumors were generally of higher histological grade and that specific morphological patterns, such as scirrhous carcinoma, were more frequent in ER/PR-positive tumors, whereas solid-tubular and papillotubular patterns were also observed in HER2-enriched cases. In contrast, our study did not identify any distinct histopathological pattern among HER2-positive tumors on the basis of HR status. Like Tamaki et al., we observed that HR-negative/HER2-positive tumors tended to have higher-grade histology, but the morphological features were heterogeneous and lacked a consistent pattern. ^19^

Hashmi et al. analyzed the demographic and pathologic features of HER2 positive and luminal HER2-positive breast carcinomas. Compared with luminal cancers, HER2-inherent breast cancers are of higher grade and have a higher Ki67 index on IHC. However, all the patients had similar TNM staging. No significant associations were noted for demographics, tumor-infiltrating lymphocytes, LVI or PNI, DCIS, or Paget’s disease. ^20^ Our study also did not reveal any association with LVI or PNI. Harish Sadashiv et al. studied 186 cases of breast carcinoma and reported that higher stages (T3, T4) were more common among patients with HER2-enriched and triple-negative subtypes (51.5% and 65.2%, respectively). The HER2-enriched subtype had a greater proportion of lymph-node metastases. However, in their study, LVI, which is associated with increased metastatic potential, was significantly greater in the luminal B (OR 4.27 95% CI 2, 9.2) and triple-negative breast carcinoma (TNBC) (OR 4.26 95% CI 2.11, 8.61) subtypes. ^21^ Puneet Somal et al analyzed 1625 cases received in a tertiary care center in North India, spread across a 6-year period. They studied clinicopathological characteristics across different molecular subtypes of breast carcinoma. They reported that Luminal B subtype was the most common subtype, accounting for approximately 42% of cases, with 17.78% of HER2-positive luminal B cases 15.69% of HER2-enriched cases, and the remaining subtypes. No significant differences were noted among the various groups with respect to age, although TNBC was present in a slightly younger age group. All cases across the molecular subtypes had a greater number of patients in the T2 stage. ^22^ This study also analyzed the clinicopathological features of two subgroups of Luminal B HER2-positive tumors: triple positive (TP) and ER-positive only. PR-positivity is known to affect overall survival (OS) and breast cancer-related survival in luminal B patients. The triple-positive subgroup shows better survival and OS than the PR-negative subgroups (ER+/PR-/HER2+ and ER+/PR-/HER2-) irrespective of HER2 status. ^23^ The absence of PR is thought to be the result of a defective ER or the overexpression of growth factor receptors, particularly EGFR. PR-negative cases are also noted to be resistant to hormonal therapy and not as responsive to NACT as HER2-enriched or triple-negative breast cancers. ^19^ Luminal B breast cancers with absence of PR expression were found to have a shorter OS and a greater association with distant metastasis, than were PR-positive patients, irrespective of HER2 status. ^20^ In the present study, the ER-positive only subgroup (group 2), was associated with worse prognostic indicators such as LVI and skin involvement, than both the TP subgroup and the HER2-enriched group, corroborating earlier findings by researchers. ^24^ In our study, HR-negative/HER2-positive tumors had a greater proportion of Grade 3 SBR histology than did HR-positive/HER2-positive tumors, which were mostly Grade 2. Other features, including LVI, PNI, DCIS, necrosis, and skin involvement, were not significantly different between the groups. Ki67 was slightly higher in HR-negative/HER2-positive tumors, reflecting a trend toward increased proliferation.

## Conclusion

This study highlights the clinicopathological heterogeneity within HER2-positive breast carcinomas when stratified by hormone receptor status. The HER2-enriched subgroup was associated with significantly older age and higher histological grade, indicating a more aggressive tumor phenotype. In contrast, the triple-positive subgroup and the ER-positive only subgroup presented lower-grade tumors and a younger age profile. While a larger tumor size, higher nodal stage was seen to be associated with HER2-enriched subtype, these were marginally significant statistically. The Ki67 index and other pathological features did not differ significantly among the groups. However, the observed variations in age and tumor grade underscore the importance of molecular subtyping for prognostication and treatment planning. Integrating hormone receptor status with HER2 positivity is essential for personalized management and outcome prediction in patients with breast cancer.

### Limitations

The *p* value might not be representative because of the low number of cases. Hence, larger study groups are recommended for appropriate values.

## Ethical considerations

The study has been approved by the Kasturba Medical College and Kasturba Hospital Institutional Ethics Committee (IEC) (Ref: IEC1:191/2024) with date of approval 18/06/2024.

## Consent

As the study is retrospective does not involve any intervention of subjects and uses lab based coded data collection; Consent waived by the ethics committee.

## Data Availability

Figshare: Histopathological and Clinical Features of HER2-Positive Breast Cancers across Hormone Receptor Subgroups: A Cross-Sectional Analysis. DOI: https://doi.org/10.6084/m9.figshare.26868448.v2 ^25^ The project contains the following underlying data: Copy of FISH POSITIVE.xlsx (Anonymised excel sheet of clinical, pathological, immunohistochemical and treatment aspects of cases and controls) Data are available under the terms of the Creative Commons Attribution 4.0 International license (CC-BY 4.0). Figshare: STROBE CHECKLIST_HER2POSITIVE BREAST CANCERS DOI: https://doi.org/10.6084/m9.figshare.30281356.v1 ^26^ Data are available under the terms of the Creative Commons Attribution 4.0 International license (CC-BY 4.0).

## References

[1] Wolff AC Somerfield MR Dowsett M : Human Epidermal Growth Factor Receptor 2 Testing in Breast Cancer: ASCO-College of American Pathologists Guideline Update. *J. Clin. Oncol.* 2023 Aug 1;41(22):3867–3872. Epub 2023 Jun 7. 10.1200/JCO.22.02864 37284804

[2] Sathishkumar K Chaturvedi M Das P : Cancer incidence estimates for 2022 & projection for 2025: Result from National Cancer Registry Programme, India. *Indian J. Med. Res.* 2022 Oct-Nov;156(4&5):598–607. 10.4103/ijmr.ijmr_1821_22 36510887 PMC10231735

[3] Perou CM Sørlie T Eisen MB : Molecular portraits of human breast tumours. *Nature.* 2000 Aug 17;406(6797):747–752. 10.1038/35021093 10963602

[4] Franchina M Pizzimenti C Fiorentino V : Low and Ultra-Low HER2 in Human Breast Cancer: An Effort to Define New Neoplastic Subtypes. *Int. J. Mol. Sci.* 2023 Aug 14;24(16):12795. 10.3390/ijms241612795 37628975 PMC10454084

[5] Vici P Pizzuti L Natoli C : Triple positive breast cancer: a distinct subtype? *Cancer Treat. Rev.* 2015 Feb;41(2):69–76. Epub 2014 Dec 19. 10.1016/j.ctrv.2014.12.005 25554445

[6] Untch M Gelber RD Jackisch C : HERA Study Team. Estimating the magnitude of trastuzumab effects within patient subgroups in the HERA trial. *Ann. Oncol.* 2008 Jun;19(6):1090–1096. Epub 2008 Feb 21. 10.1093/annonc/mdn005 18296421

[7] Anderson WF Chatterjee N Ershler WB : Estrogen receptor breast cancer phenotypes in the Surveillance, Epidemiology, and End Results database. *Breast Cancer Res. Treat.* 2002 Nov;76(1):27–36. 10.1023/a:1020299707510 12408373

[8] Rakha EA El-Sayed ME Green AR : Biologic and clinical characteristics of breast cancer with single hormone receptor positive phenotype. *J. Clin. Oncol.* 2007 Oct 20;25(30):4772–4778. Epub 2007 Sep 17. 10.1200/JCO.2007.12.2747 17876012

[9] Asif HM Sultana S Ahmed S : HER-2 Positive Breast Cancer - a Mini-Review. *Asian Pac. J. Cancer Prev.* 2016;17(4):1609–1615. 10.7314/apjcp.2016.17.4.1609 27221828

[10] Ran R Zhao S Zhou Y : Clinicopathological characteristics, treatment patterns and outcomes in patients with HER2-positive breast cancer based on hormone receptor status: a retrospective study. *BMC Cancer.* 2024 Sep 30;24(1):1216. 10.1186/s12885-024-12974-4 39350043 PMC11443648

[11] Swain SM Shastry M Hamilton E : Targeting HER2-positive breast cancer: advances and future directions. Nat. Rev. Drug Discov. 2023 Feb;22(2):101–126. 10.1038/s41573-022-00579-0 36344672 PMC9640784

[12] Da Ros L Moretti A Querzoli P : HER2-Positive Lobular Versus Ductal Carcinoma of the Breast: Pattern of First Recurrence and Molecular Insights. *Clin. Breast Cancer.* 2018 Oct;18(5):e1133–e1139. Epub 2018 Apr 18. 10.1016/j.clbc.2018.04.006 29759595

[13] Skenderi F Alahmad MAM Tahirovic E : HER2-positive apocrine carcinoma of the breast: a population-based analysis of treatment and outcome. Breast Cancer Res. Treat. 2022 Jun;193(2):523–533. 10.1007/s10549-022-06578-4 35355162 PMC9090698

[14] Vranic S Tawfik O Palazzo J : EGFR and HER-2/neu expression in invasive apocrine carcinoma of the breast. *Mod. Pathol.* 2010 May;23(5):644–653. Epub 2010 Mar 5. 10.1038/modpathol.2010.50 20208479

[15] Akashi M Yamaguchi R Kusano H : Diverse histomorphology of HER2-positive breast carcinomas based on differential ER expression. *Histopathology.* 2020 Mar;76(4):560–571. Epub 2020 Feb 3. 10.1111/his.14003 31554015

[16] Taucher S Rudas M Mader RM : Do we need HER-2/neu testing for all patients with primary breast carcinoma? *Cancer.* 2003 Dec 15;98(12):2547–2553. 10.1002/cncr.11828 14669272

[17] Omranipour R Nazarian N Alipour S : Evaluation of HER2 Positivity Based on Clinicopathological Findings in HER2 Borderline Tumors in Iranian Patients with Breast Cancer. *Iran. J. Pathol.* 2023;18(4):403–409. Epub 2023 Oct 15. 10.30699/IJP.2023.561915.2970 38024548 PMC10646737

[18] Dang Cong T Nguyen Thanh T Nguyen Phan QA : Correlation between HER2 Expression and Clinicopathological Features of Breast Cancer: A Cross- Sectional Study in Vietnam. *Asian Pac. J. Cancer Prev.* 2020 Apr 1;21(4):1135–1142. 10.31557/APJCP.2020.21.4.1135 32334482 PMC7445976

[19] Tamaki M Kamio T Kameoka S : The relevance of the intrinsic subtype to the clinicopathological features and biomarkers in Japanese breast cancer patients. *World J. Surg. Oncol.* 2013 Nov 18;11:293. 10.1186/1477-7819-11-293 24245483 PMC3913623

[20] Hashmi AA Mahboob R Khan SM : Clinical and prognostic profile of Her2neu positive (non-luminal) intrinsic breast cancer subtype: comparison with Her2neu positive luminal breast cancers. *BMC. Res. Notes.* 2018 Aug 13;11(1):574. 10.1186/s13104-018-3677-y 30103802 PMC6090780

[21] Harish S Anand S Prashar M : Intrinsic subtyping of breast cancer and its relevance with clinico-pathological features and outcomes in patients from North India: a single center experience. *Journal of Dr. NTR University of Health Sciences.* Jul–Sep 2020;9(3):164–171. 10.4103/JDRNTRUHS.JDRNTRUHS_77_20

[22] Somal PK Sancheti S Sharma A : A Clinicopathological Analysis of Molecular Subtypes of Breast Cancer using Immunohistochemical Surrogates: A 6-Year Institutional Experience from a Tertiary Cancer Center in North India. *South Asian J. Cancer.* 2023 Mar 9;12(2):104–111. 10.1055/s-0043-1761942 37969672 PMC10635761

[23] Cancello G Maisonneuve P Rotmensz N : Progesterone receptor loss identifies Luminal B breast cancer subgroups at higher risk of relapse. *Ann. Oncol.* 2013 Mar;24(3):661–668. Epub 2012 Sep 28. 10.1093/annonc/mds430 23022996

[24] Prat A Cheang MC Martín M : Prognostic significance of progesterone receptor-positive tumor cells within immunohistochemically defined luminal A breast cancer. *J. Clin. Oncol.* 2013 Jan 10;31(2):203–209. Epub 2012 Dec 10. 10.1200/JCO.2012.43.4134 23233704 PMC3532392

[25] Prabhu SC : Histopathological and Clinical Features of HER2-Positive Breast Cancers across Hormone Receptor Subgroups: A Cross-Sectional Analysis.Dataset. *figshare.* 2024. 10.6084/m9.figshare.26868448.v2 PMC12982978 41835980

[26] Prabhu SC : STROBE CHECKLIST_HER2 POSITIVE BREAST CANCERS.Dataset. *figshare.* 2025. 10.6084/m9.figshare.30281356.v1

