# Supplementary material for: Histopathological and Clinical Features of HER2-Positive Breast Cancers across Hormone Receptor Subgroups: A Cross-Sectional Analysis
Source: F1000Res. 2025 Nov 13;14:1246. [Version 1] doi: 10.12688/f1000research.171847.1 (PMC12982978; doi:10.12688/f1000research.171847.1)
Supplement: Peer review history

# Reviewer report: Reviewer response for version 1

**DOI:** 10.5256/f1000research.189505.r454043

**Published:** 2026-03-12

**Authors:** Archana Bhat

**Affiliations:** 1 Father Muller Medical College, Mangaluru, Karnataka, India

The authors have studied HER2-positive breast carcinomas and compared the clinicopathological features across three subgroups: Triple-positove, ER-positive only, and HER2-enriched groups.

 They observed statistically significant differences in the mean age (p = 0.001) and SBR score (p = 0.004) among the three groups. However, there was no significant difference in tumor size (p = 0.143) or the Ki67 index (p = 0.400) between the groups. Post hoc analysis revealed a significant difference in age between group 1 and group 3 (p = 0.001), with group 3 being older. Similarly, SBR scores were significantly higher in group 3 than in group 1 (p = 0.004).

 However, the authors mention no details on the method of Ki67 evaluation (? eyeballing method) which may impact the results as majority of the cases (83.8%) included are core-biopsies.

 Another concern is regarding the assessment of lymphovascular invasion, perineural invasion, DCIS, in the core biopsy specimens, which may not be entirely representative as compared to BCS samples (8.6%) and MRM (7.6%) of the total samples. 

 The statistically significant association for SBR grade but not for Ki67 needs to be explored further to check if HER2 status has any association with glandular/ tubule formation.  

 This study is a good attempt to highlight the clinicopathologic heterogeneity within HER2-positive breast carcinomas and subgroups.

Is the work clearly and accurately presented and does it cite the current literature?

Yes

If applicable, is the statistical analysis and its interpretation appropriate?

Partly

Are all the source data underlying the results available to ensure full reproducibility?

Yes

Is the study design appropriate and is the work technically sound?

Partly

Are the conclusions drawn adequately supported by the results?

Partly

Are sufficient details of methods and analysis provided to allow replication by others?

Yes

Reviewer Expertise:

Renal Pathology, Cytopathology, Medical education, Pulmonary Pathology, Oncopathology

I confirm that I have read this submission and believe that I have an appropriate level of expertise to confirm that it is of an acceptable scientific standard.

## References

[1] : The relevance of the intrinsic subtype to the clinicopathological features and biomarkers in Japanese breast cancer patients. *World Journal of Surgical Oncology* .2013;11(1) : 10.1186/1477-7819-11-293 10.1186/1477-7819-11-293 24245483 PMC3913623

# Reviewer report: Reviewer response for version 1

**DOI:** 10.5256/f1000research.189505.r454044

**Published:** 2026-02-02

**Authors:** Prema Saldanha

**Affiliations:** 1 Department of Pathology, Yenepoya Medical College, Deralakatte, Karnataka, India

Identification of HER2-positive breast cancers is crucial due to the availability of targeted therapies. However, these tumours exhibit significant clinico-pathological heterogeneity, variation in hormone receptor status and differences in gene expression profiles. This study by Rao ACK, et al, has identified the clinical and histopathological characteristics in the three subgroups based on the hormone receptor status. This knowledge is important for prognostication as well as for guiding the selection of treatment modalities in individual patients.

Is the work clearly and accurately presented and does it cite the current literature?

Yes

If applicable, is the statistical analysis and its interpretation appropriate?

Yes

Are all the source data underlying the results available to ensure full reproducibility?

Yes

Is the study design appropriate and is the work technically sound?

Yes

Are the conclusions drawn adequately supported by the results?

Yes

Are sufficient details of methods and analysis provided to allow replication by others?

Yes

Reviewer Expertise:

Histopathology

I confirm that I have read this submission and believe that I have an appropriate level of expertise to confirm that it is of an acceptable scientific standard.

## References

[1] : Histopathological and Clinical Features of HER2-Positive Breast Cancers across Hormone Receptor Subgroups: A Cross-Sectional Analysis. *F1000Research* .2025;14: 10.12688/f1000research.171847.1 10.12688/f1000research.171847.1 40657179
